# Left Atrial Deformation Analysis in Patients with Corrected Tetralogy of Fallot by 3D Speckle-Tracking Echocardiography (from the MAGYAR-Path Study)

**DOI:** 10.5935/abc.20170004

**Published:** 2017-02

**Authors:** Kálmán Havasi, Péter Domsik, Anita Kalapos, Jackie S. McGhie, Jolien W. Roos-Hesselink, Tamás Forster, Attila Nemes

**Affiliations:** 1 2nd Department of Medicine and Cardiology Center - Medical Faculty - Albert Szent-Györgyi Clinical Center - University of Szeged - Szeged - Hungary; 2 Department of Cardiology - Erasmus MC - Rotterdam - The Netherlands

**Keywords:** Echocardiography, Three-Dimensional / methods, Heart Atria / abnormalities, Tetralogy of Fallot, Heart Defects, Congenital

## Abstract

**Background:**

Three-dimensional (3D) echocardiography coupled with speckle-tracking echocardiographic (STE) capability is a novel methodology which has been demontrated to be useful for the assessment of left atrial (LA) volumes and functional properties. There is increased scientific interest on myocardial deformation analysis in adult patients with corrected tetralogy of Fallot (cTOF).

**Objectives:**

To compare LA volumes, volume-based functional properties and strain parameters between cTOF patients and age- and gender-matched healthy controls.

**Methods:**

The study population consisted of 19 consecutive adult patients with cTOF in sinus rhythm nursing at the University of Szeged, Hungary (mean age: 37.9 ± 11.3 years, 8 men, who had repair at the age of 4.1 ± 2.5 years). They all had undergone standard transthoracic two-dimensional Doppler echocardiographic study extended with 3DSTE. Their results were compared to 23 age- and gender-matched healthy controls (mean age: 39.2 ± 10.6 years, 14 men).

**Results:**

Increased LA volumes and reduced LA emptying fractions respecting cardiac cycle could be demonstrated in cTOF patients compared to controls. LA stroke volumes featuring all LA functions showed no differences between the 2 groups examined. LA global and mean segmental uni- and multidirectional peak strains featuring LA reservoir function were found to be diminished in adult patients with cTOF as compared to controls. Similarly to peak strains reduced global and mean segmental LA strains at atrial contraction characterizing atrial booster pump function could be demonstrated in cTOF patients as compared to controls.

**Conclusions:**

Significant deterioration of all LA functions could be demonstrated in adult patients with cTOF late after repair.

## Introduction

Nowadays the angle-independent speckle-tracking echocardiography-derived (STE) myocardial deformation analysis is one of the main focus of cardiac ultrasound technology.^1^ Three-dimensional (3D) echocardiography coupled with STE capability is a novel methodology which has been demonstrated to be useful for the assessment of volumes and functional properties of cardiac chambers.^2^ 3DSTE allows complex assessment of atrial and ventricular morphology and function including volumetric and strain measurements from the same acquired 3D dataset.

There is increased scientific interest on myocardial deformation analysis in adult patients with corrected tetralogy of Fallot (cTOF).^3-5^ Recently, alterations in right (RV)^3,4^ and left ventricular (LV)^4^ and right atrial (RA)^5^ functional properties could be demonstrated by 3DSTE. However, quantitative left atrial (LA) deformation assessment has never been performed in cTOF patients. Therefore, the present study aimed to detect changes in LA volumes, volume-based functional properties and strain parameters in cTOF patients as compared to age- and gender-matched healthy controls.

## Methods

### Patient population

Since 1961, more than 2,700 congenital heart disease patients have been treated and/or operated on at the Department of Pediatrics, Department of Heart Surgery, and 2nd Department of Medicine and Cardiology Center at the University of Szeged. From this patient population a registry was created (CSONGRAD Registry),^6^ from which 19 consecutive adult patients with cTOF in sinus rhythm were willing to participate in the present study (mean age: 37.9 ± 11.3 years, 8 men) who had repair at the age of 4.1 ± 2.5 years. In our department several hundreds of healthy control subjects without risk factors or known disorders in different age groups were examined by 3DSTE to assess the normal values of 3DSTE-derived parameters. From this pool 20 age- and gender-matched healthy subjects (mean age: 39.2 ± 10.6 years, 14 men) were selected who served as a control group in this particular study. All cTOF patients and controls were examined by two-dimensional (2D) Doppler, tissue Doppler echocardiography (TDI) and 3DSTE. The present study is a part of **MAGYAR-Path Study** (**M**otion **A**nalysis of the heart and **G**reat vessels b**Y** three-dimension**A**l speckle-t**R**acking echocardiography in **Path**ological cases) which has been organized at our department to examine diagnostic and prognostic significance of 3DSTE-derived variables. The institutional human research committee approved the study which complied with the 1975 Declaration of Helsinki. Informed consent was obtained from all cTOF patients and control subjects.

### Two-dimensional Doppler and tissue Doppler echocardiography

All M-mode (MME), 2D Doppler and TDI studies were performed in the left lateral decubitus position with a commercially available Toshiba Artida^™^ echocardiography equipment (Toshiba Medical Systems, Tokyo, Japan) using a PST-30SBP phased-array transducer in all patients. LV dimensions were assessed by MME using the Teichholz method.^7^ Valvular regurgitations were confirmed by colour Doppler echocardiography-derived visual grading. Following Doppler assessment of E/A, the ratio of transmitral E velocity to early diastolic mitral annular velocity (E/E') was measured by TDI.

### Three-dimensional speckle-tracking echocardiography

All 3D echocardiographic data acquisitions were performed using a 1-4 MHz PST-25SX matrix phased-array transducer (Toshiba Medical Systems, Tokyo, Japan).^2^ During a single breathhold full volume 3D datasets were created from the apical view from 6 wedge-shaped subvolumes using 6-beat electrocardiographically gated acquisitions. LA was quantified by the 3D Wall Motion Tracking software version 2.7 (Toshiba Medical Systems, Tokyo, Japan).^8^ Each 3D dataset was displayed in a five plane-view, namely apical two- (AP2CH) and four-chamber (AP4CH) views and three short-axis views at different levels of the LA. After positioning the main axis line through the center of the LA cavity the reader traced LA endocardial border in both orthogonal long-axis views. Firstly, the edge of the septal side of the mitral valve ring was traced, then markers were set in a counterclockwise rotation around the LA to the edge of the lateral side of the mitral valve ring. Subsequently, 3D wall motion tracking was then automatically performed through the entire cardiac cycle.

### 3DSTE for left atrial volumetric measurements

**Figure 1:**
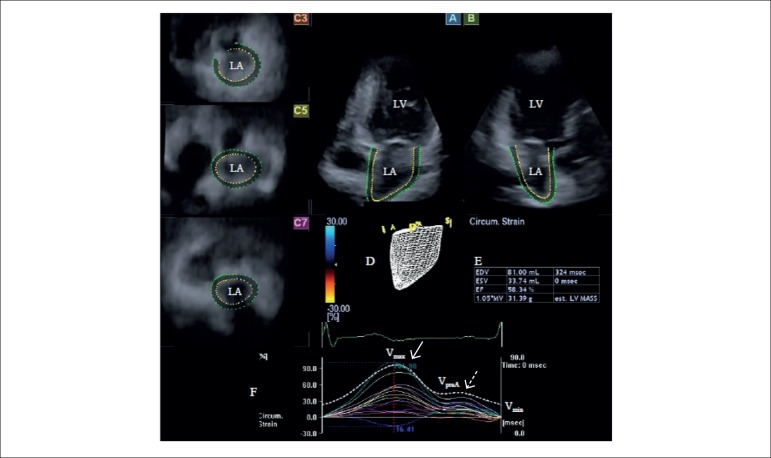
- Images from three-dimensional (3D) full-volume dataset showing left atrium (LA) in a patient with corrected tetralogy of Fallot is presented: (A) apical four-chamber view, (B) apical two-chamber view, (C3) short-axis view at basal, (C5) mid- and (C7) superior left atrial level. A 3D cast (D), volumetric data (E), time - global volume and time - segmental strain curves (F) of the LA are also presented. Dashed curve (F) represents LA volume changes during cardiac cycle with maximum (V_max_), minimum (V_min_) LA volumes and LA volume at atrial contraction (V_preA_). White arrow represents peak strain, while dashed arrow represents strain at atrial contraction (F). LA: left atrium; LV: left ventricle.

To characterize systolic reservoir and diastolic conduit and active contraction phases of the LA function, calculation of volume-based functional properties respecting cardiac cycle is an available option (Figure 1).^8-12^ End-systolic LA volume [largest LA volume before mitral valve opening (V_max_)], end-diastolic LA volume [smallest LA volume before mitral valve closure (V_min_)] and diastolic LA volume before atrial contraction [at time of P wave on ECG (V_preA_)] could be measured using the LA 3D cast, from which the following functional properties were calculated:

Reservoir function:

- Total Atrial Stroke Volume (TASV): V_max_−V_min_.
- Total Atrial Emptying Fraction (TAEF): TASV/V_max_×100.

Conduit function:

- Passive Atrial Stroke Volume (PASV): V_max_−V_preA_
- Passive Atrial Emptying Fraction (PAEF): PASV/V_max_×100

Active contraction:

- Active Atrial Stroke Volume (AASV): V_preA_−V_min_.
- Active Atrial Emptying Fraction (AAEF): AASV/V_preA_×100.

### 3DSTE for left atrial strain measurements

Several unidirectional [radial (RS), longitudinal (LS) and circumferential (CS) strains] and complex [area (AS) and 3D (3DS) strains] LA strain parameters could be calculated from the same 3D model, as demonstrated before.^10-14^ Not only global and mean segmental peak strains featuring LA reservoir function were measured for each patient, but strains at atrial contraction, characteristics of LA active contraction, were also evaluated (Figure 1).

### Stastical analysis

Continuous data are presented as mean values ± standard deviation, while categorical data are summarized as a count and percentage. For comparing variables, the Student's *t-*test, chi-square analysis, and Fisher's exact test were used. All statistical tests were two-tailed and statistical significance was defined with a probability value less than 0.05. Recently, intra- and interobserver agreements for LA volumes and functional properties were performed in papers originating from MAGYAR-Healthy and MAGYAR-Path Studies.^8,11^ Data were analysed using Medcalc software (MedCalc, Mariakerke, Belgium).

## Results

### Clinical data

Risk factors, medications applied and 2D echocardiographic data are presented in Table 1. Significant (>grade 2) mitral and tricuspid regurgitations could be detected in 2 (11%) and 8 (42%) cTOF patients. None of the healthy controls had significant regurgitations. The TAPSE and RV-FAC values of cTOF patients proved to be 18.2 ± 4.6 mm and 34.2 ± 3.9%, respectively.

**Table 1:** Demographic and clinical data of patients with tetralogy of Fallot and that of controls

	cTOF patients (n=19)	Controls (n=23)	p value
**Risk factors**			
Age (years)	37.9 ± 11.3	39.2 ± 10.6	0.70
Male gender (%)	8 (42)	14 (61)	0.35
Hypertension (%)	3 (16)	0 (0)	0.08
Hypercholesterolemia (%)	1 (5)	0 (0)	0.45
Diabetes mellitus (%)	0 (0)	0 (0)	1.00
**Medications**			
β-blockers (%)	5 (26)	0 (0)	0.01
ACE-inhibitors (%)	3 (16)	0 (0)	0.08
Diuretics (%)	3 (16)	0 (0)	0.08
**Two-dimensional echocardiography**			
LA diameter (mm)	42.4 ± 6.8	33.2 ± 3.8	<0.0001
LV end-diastolic diameter (mm)	54.6 ± 19.6	48.3 ± 6.9	0.16
LV end-diastolic volume (ml)	113.7 ± 31.7	102.2 ± 21.1	0.17
LV end-systolic diameter (mm)	32.7 ± 7.1	30.4 ± 4.1	0.20
LV end-systolic volume (ml)	43.8 ± 23.2	35.6 ± 10.6	0.14
Interventricular septum (mm)	9.9 ± 1.5	9.5 ± 2.0	0.46
LV posterior wall (mm)	9.8 ± 1.5	9.4 ± 2.3	0.55
LV ejection fraction (%)	62.7 ± 11.5	65.4 ± 6.5	0.34

ACE: angiotensin-converting enzyme; LA: left atrial; LV: left ventricular; cTOF: corrected tetralogy of Fallot.

### 3DSTE-derived LA volumes and volume-based functional properties

Increased LA volumes and reduced LA emptying fractions respecting cardiac cycle could be demonstrated in cTOF patients compared to controls. LA stroke volumes featuring all LA functions showed no differences between the groups examined (Table 2).

**Table 2:** Comparison of 3DSTE-derived volumes and volume-based functional properties between patients with corrected tetralogy of Fallot and controls

	Calculated volumes (ml)			Stroke volumes (ml)			Emptying fractions (%)		
	V_max_	V_min_	V_preA_	TASV	PASV	AASV	TAEF	PAEF	AAEF
cTOF patients	53.3 ± 28.1	35.1 ± 24.4	42.7 ± 26.0	18.2 ± 7.4	10.6 ± 6.4	7.6 ± 4.4	37.1 ± 11.7	21.4 ± 11.6	20.1 ± 10.8
Controls	36.8 ± 6.6	18.2 ± 6.3	26.3 ± 8.1	18.6 ± 4.1	10.5 ± 4.6	8.1 ± 3.2	51.4 ± 11.4	29.5 ± 13.3	31.1 ± 9.1
p value	0.009	0.003	0.006	0.84	0.96	0.71	0.0003	0.04	0.0009

V_max_: maximum left atrial volume; Vmin: minimum left atrial volume; V_preA_: left atrial volume before atrial contraction; TASV: total atrial stroke volume; TAEF: total atrial emptying fraction; AASV: active atrial stroke volume; AAEF: active atrial emptying fraction; PASV: passive atrial stroke volume; PAEF: passive atrial emptying fraction. cTOF: corrected tetralogy of Fallot.

### 3DSTE-derived LA peak strain parameters

LA global and mean segmental uni- and multidirectional peak strains featuring LA reservoir function were found to be diminished in adult patients with cTOF as compared to controls (Table 3).

**Table 3:** Comparison of 3DSTE-derived peak strains and strains at atrial contraction between patients with tetralogy of Fallot and controls (global and mean segmental parameters)

	Radial strain (%)		Circumferential strain (%)		Longitudinal strain (%)		Three-dimensional strain (%)		Area strain (%)	
	Global	Mean segmental	Global	Mean segmental	Global	Mean segmental	Global	Mean segmental	Global	Mean segmental
**Peak strains**										
cTOF patients	-12.8 ± 9.5	-17.0 ± 8.5	13.2 ± 9.2	18.3 ± 8.8	17.4 ± 8.3	19.7 ± 8.1	-7.0 ± 6.3	-11.5 ± 6.2	33.1 ± 14.2	38.9 ± 13.7
Controls	-18.0 ± 9.9	-21.7 ± 8.9	29.0 ± 13.4	34.2 ± 13.1	26.3 ± 7.7	29.6 ± 7.4	-11.0 ± 8.2	-15.1 ± 6.9	59.7 ± 22.0	67.9 ± 21.7
p value	0.10	0.09	0.0001	0.0001	0.0008	0.0002	0.09	0.09	0.0001	<0.0001
**Strains at atrial contraction**										
cTOF patients	-2.8 ± 4.6	-6.5 ± 5.6	4.5 ± 5.0	7.3 ± 5.0	2.9 ± 4.6	4.7 ± 3.9	-1.7 ± 6.4	-4.7 ± 4.8	8.1 ± 9.7	12.4 ± 8.9
Controls	-7.2 ± 7.9	-8.2 ± 5.5	11.2 ± 10.4	13.9 ± 9.2	8.1 ± 8.8	9.0 ± 5.8	-5.5 ± 5.1	-6.4 ± 4.8	16.7 ± 16.1	20.3 ± 14.2
p value	0.03	0.33	0.01	0.008	0.03	0.10	0.04	0.24	0.04	0.04

cTOF: corrected tetralogy of Fallot.

### 3DSTE-derived LA strain parameters at atrial contraction

Similarly to peak strains reduced global and mean segmental LA strains at atrial contraction characterizing atrial booster pump function could be demonstrated in cTOF patients as compared to controls (Table 3).

## Discussion

Three-dimensional speckle-tracking echocardiography, which is an echocardiographic technique based on block-matching algorithm of the myocardial speckles,^2^ has been increasingly used as a tool for volumetric and functional assessment of atria^5,8-14^ and ventricles.^3,15-21^ In recent studies 3DSTE-derived complex evaluation of LA function including assessment of volume-based functional properties and strains has been demonstrated.^8-14^ The study reported here is the first to analyse 3DSTE-derived LA deformation in adult patients with cTOF. Increased LA volumes and diminished LA emptying fractions and strains could be demonstrated in this detailed analysis. Results suggest significant deterioration of all LA functions (reservoir, conduit and booster pump) in adult patients with cTOF late after repair.

STE was found to be a valuable tool for volumetric and functional assessment of cardiac chambers in adult patients with cTOF.^3-5^ In a recent study RV free wall strain and strain rate were found to be decreased in adults late after TOF repair, especially at the apical segment suggesting that apical function is most affected in these RVs.^4^ Regarding the LV, septal strain was decreased indicating that RV dysfunction adversely affects LV function, probably by mechanical coupling of the ventricles. In another study, the majority of adults with cTOF showed a reduced LV twist.^22^ Strikingly, one-quarter of these patients had an abnormal apical rotation which has been found to be associated with decreased systolic LV and RV function. These findings suggested that abnormal apical rotation could be a new objective diagnostic criterion for detection of ventricular dysfunction in cTOF.

The complexity of RA dysfunction could also be demonstrated by 3DSTE in cTOF patients.^5^ Comparing this with the present study, both RA and LA volumes seemed to be increased in adult patients with cTOF. Moreover, large similarity of RA and LA deformation could also be demonstrated: while RA/LA emptying fractions were found to be decreased, RA/LA stroke volumes remained unchanged. All the peak LA strains and LA strains at atrial contraction were found to be reduced and this reduction was more pronounced in cTOF as compared to the values related to RA. Therefore it seems that the LA is very important. From other studies also the LA proved to be important.^23^

Several factors may play a role in the altered atrial function in cTOF, such as the interaction between both atria, the presence of mitral/tricuspid regurgitation, arrhythmias and changes in both ventricular features as demonstrated before. Further studies are warranted to understand the real pathophysiologic background of these findings.

### Limitation

The present study covered only a relatively small number of patients from a single center by a single observer (DP). Therefore, future multicenter studies with larger patient populations are necessary. Another limitation of image acquisition for 3DSTE is the relatively slow volume rate. During creating 3D model of the LA, septum was considered as a part of the LA similarly to other studies evaluating RA.^5^ Finally, LA appendage and pulmonary veins were excluded which could theoretically affect results. During the present study, LV, RV and RA functional characterization was not aimed to be performed.

## Conclusions

Significant deterioration of all LA functions could be demonstrated in adult patients with cTOF late after repair.
